# Antiseptic Surgical Dressing

**Published:** 1898-09

**Authors:** 


ANTISEPTIC SURGICAL DRESSING. ℞.—Extract eucalyptus, fl., 2 parts; spt. vini rect., 6 parts. M. An ointment consisting of oil of eucalyptus 1 part and vaseline 4 parts makes an excellent dressing for all forms of skin diseases.

